# *Sergentomyia (Neophlebotomus) gemmea*, a potential vector of *Leishmania siamensis* in southern Thailand

**DOI:** 10.1186/1471-2334-13-333

**Published:** 2013-07-19

**Authors:** Kobkan Kanjanopas, Suradej Siripattanapipong, Ubolrat Ninsaeng, Atitaya Hitakarun, Somnat Jitkaew, Preecha Kaewtaphaya, Peerapan Tan-ariya, Mathirut Mungthin, Chetsuda Charoenwong, Saovanee Leelayoova

**Affiliations:** 1 Ministry of Public Health, Nonthaburi Province 11000, Thailand; 2 Department of Parasitology, Phramongkutklao College of Medicine, 315 Ratchawithi Road, Ratchathewi, Bangkok 10400, Thailand; 3 The Vector-borne Disease Control Center 11.3, Ministry of Public Health, Suratthani Province, Thailand; 4 Faculty of Science, Mahidol University, Bangkok, Thailand; 5 The Vector-borne Disease Control Center 12.3, Ministry of Public Health, Trang Province, Thailand; 6 Yantakao Hospital, Trang Province, Thailand

**Keywords:** Leishmaniasis, *Leishmania siamensis*, *Sergentomyia* (*Neophlebotomus*) *gemmea*, Vector, Thailand

## Abstract

**Background:**

Leishmaniasis, caused by *Leishmania siamensis*, is an emerging disease in Thailand. Although reported cases have been increasing, epidemiological information of the disease including host and vector aspects is not clearly known. This study was a preliminary survey of the potential vector of *L. siamensis* in an affected area of leishmaniasis, Trang Province, southern Thailand.

**Methods:**

The collection of sandflies was performed around the area where a case of leishmaniasis was reported using CDC light traps. Species of sandfly were identified based on morphological characteristics according to Lewis’s key. PCR amplification and sequencing of the heat shock protein 70 gene (*hsp*70) was used to identify *L. siamensis* DNA in sandflies.

**Results:**

A total of 146 male and female sandflies were collected in the affected areas. Of 71 female sandflies, four species were identified, i.e., *Sergentomyia* (*Neophlebotomus*) *gemmea*, *S.* (*Neophlebotomus*) *iyengari, S. (Parrotomyia) barraudi* and *Phlebotomus (Anaphlebotomus) stantoni*. Among these species, *S.* (*Neophlebotomus*) *gemmea* was the most predominant species in all areas. DNA of *L. siamensis* was identified in *S.* (*Neophlebotomus*) *gemmea.* Nucleotide sequences of PCR products using DNA extracted from *S.* (*Neophlebotomus*) *gemmea* showed 99.8% identity to *L. siamensis.*

**Conclusion:**

*S.* (*Neophlebotomus*) *gemmea* might be a potential vector of *L. siamensis* in an affected area, Trang Province, southern Thailand. However further studies are needed to prove whether these sandflies can be natural vectors of leishmaniasis.

## Background

Autochthonous visceral leishmaniasis (VL) caused by *Leishmania siamensis*, firstly described from southern Thailand in 2008, is now considered an emerging disease in Thailand [1]. Previously, only two autochthonous leishmaniasis cases were reported in Thailand, i.e., in 1999 and 2007; however, the causative species were not identified [2,3]. After 2008, more cases of autochthonous leishmaniasis were reported [4-6]. Characterization of the causative species, *L. siamensis* was performed and showed that *L. siamensis* is a novel species [4,7]. HIV/AIDS patients are a high risk group to acquire the infection which can cause not only VL but also disseminated dermal leishmaniasis [4-6]. Until now, the disease has been reported from six provinces in the South, two provinces in the North as well as one province in the East of Thailand [4,6]. Zoonotic transmission of *L. siamensis* could possibly occur since animal cutaneous leishmaniasis caused by *L. siamensis* was recently reported from Switzerland, Germany and the USA [8-10]. Identification of the vectors is crucial for the prevention and control of the disease. Surveys of sandfly species have been conducted in the Central, West, North, Northeast and South of Thailand where *Sergentomyia* is the most predominant genus reported in all study areas [1,11-14], of which *S.* (*Neophlebotomus*) *gemmea* is the most prevalent in the South [14]. However, the particular species of sandfly that could serve as the vector for leishmaniasis has not yet been demonstrated.

## Methods

In 2011, a sentinel surveillance of sandfly species distribution and their potential vectors was conducted in an affected area, Hat Samran District, Trang Province, southern Thailand where a case of VL-HIV co-infection was reported. The altitude of the affected area is 18 m above the sea level where palm trees were grown in the area. In August 2011, the average temperature was 27.9°C with a humidity of 85.5%. The average wind speed was 1.3 knots and the rainfall was 10.0 mm. Outdoors, a 200-m perimeter surrounding the house included animal enclosures, i.e., cows, goats, pigs, dogs, and Persian cats was surveyed. Neighborhood houses were about 10 m apart. A palm tree plantation was located about 50 m away from the house.

To capture sandflies, CDC light traps were placed indoors and outdoors of the patient’s house in August, 2011 from 18.00-06.00 h. Outdoors, areas of the 200-m perimeter surrounded house included areas behind the house, spaces under the kitchen, animal enclosures, stacks of bricks and wood in the back yard, banana plants, palm trees, a store house for wood and rice, a thatched roof hut and a nearby abandoned house.

Species of female sandflies were identified by well-trained entomologists based on morphological characteristics [15,16] at the Vector-borne Disease Control Center 11.3, Suratthani Province, Thailand. These female sandflies were pooled in the same microcentrifuge tube containing 70% alcohol if they shared the same characteristics, i.e., species and collected place. The samples were then sent to the Department of Parasitology, Phramongkutklao College of Medicine for molecular studies. DNA from each pool was extracted using the Genomic DNA Mini Kit (Tissue) (Geneaid, Taiwan) and evaluated for natural infections with *L. siamensis* using PCR amplification of the heat shock protein 70 gene (*hsp*70) and the internal transcribed spacer 1 region (ITS1) of the small subunit ribosomal RNA (SSU-rRNA) gene. For the *hsp*70, PCR was performed using primers HSP70sen and HSP70ant with the conditions previously described [17]. For the ITS1, the method of El Tai et al. (2001) was performed [18]. DNA sequencing was then conducted by 1st Base Pte. Ltd., Singapore. All PCR products positive for *hsp70* gene were ligated with pGEM-T Easy vector (Promega, WI, USA) and cloned into *E.coli* JM109. Three clones of each positive pool were selected and sequenced.

The 18s rRNA region of each pool sandfly sample was amplified using primers Lu.18S rRNA-1S and Lu.18S rRNA-1R with conditions previously described by Kato et al. [19]. The PCR products from each pool were then directly sequenced.

The minimum infection rate (MIR) was calculated using the standard formula: [number of positive pools / total specimens tested] x 1000), with the data representing a single sandfly species, collected over a time period and geographic area.

## Results

A total of 146 captured sandflies were collected with a female:male ratio of 1:1.1. Of 71 female sandflies, four species were identified of which *Sergentomyia* (*Neophlebotomus*) *gemmea* (49.3%) was the most predominant species, followed by *S.* (*Neophlebotomus*) *iyengari* (42.3%)*, S. (Parrotomyia) barraudi* (4.2%) and *Phlebotomus (Anaphlebotomus) stantoni* (4.2%) (Table 1). The most common habitat of sandflies was at the cow corral (39.4%) followed by the thatched roof hut (26.8%). Others were the chicken stall (19.7%), the store house (7.1%), banana plants (4.2%) and the abandoned house (2.8%). *S.* (*Neophlebotomus*) *gemmea* was found at all mentioned habitats, mostly at the cow corral and the thatched roof hut. None were trapped indoors.

**Table 1:** Species and habitats of trapped female sandflies in the study area

**Sandfly species**	**Areas of trapped sandflies**						
	**Cow corral**	**Chicken stall**	**Banana plants**	**Thatched roof hut**	**Woodshed**	**Abandoned house**	**Total**
*Phlebotomus (Anaphlebotomus) stantoni*	2	-	-	-	-	1	3 (4.2%)
*Sergentomyia* (*Neophlebotomus*) *gemmea*	17	1	2	11	3	1	35 (49.3%)
*Sergentomyia* (*Neophlebotomus*) *iyengari*	7	12	1	8	2	-	30 (42.3%)
*Sergentomyia (Parrotomyia) barraudi*	2	1	-	-	-	-	3 (4.2%)
Total	28 (39.4%)	14 (19.7%)	3 (4.2%)	19 (26.8%)	5 (7.1%)	2 (2.8%)	71 (100%)

All sequencing results from the cloning of positive *hsp70* amplification showed identical amplicon sizes at 1,422 bp using extracted DNA of trapped *S.* (*Neophlebotomus*) *gemmea*, which showed 99.8% identity to the *hsp70* of *L. siamensis* (GenBank accession number JX852709). GenBank accession numbers of the representative nucleotide sequences of *L. siamensis* detected in *S.* (*Neophlebotomus*) *gemmea* were assigned as JX852708. Positive samples were from two pools of *S.* (*Neophlebotomus*) *gemmea* females; the first pool was trapped at the cow corral near the house (10 females) and the other was collected at the store house (3 females). Unfortunately, we could not amplify the ITS1 from these samples. Determining the 18s rRNA of sandflies showed the consensus among nucleotide sequences from these *L. siamensis-* positive *S.* (*Neophlebotomus*) *gemmea* samples. One of these sequences was submitted to GenBank under accession number KF112045. The minimum infection rate (MIR) was 57.1 since there were two *L. siamensis*-positive pools and a total of 35 collected *S.* (*Neophlebotomus*) *gemmea*.

## Discussion

In the present study, we reported *S.* (*Neophlebotomus*) *gemmea* as a potential vector of leishmaniasis caused by *L. siamensis* in Thailand. PCR amplification and sequence analysis of the *hsp*70 gene demonstrated *L. siamensis* DNA in *S.* (*Neophlebotomus*) *gemmea* captured in the affected area where a case of VL-HIV co-infection was reported. Unfortunately, the infection rate of *S.* (*Neophlebotomus*) *gemmea* could not be determined since we used the DNA of pooled samples. To date, no report has identified the possible role of *S.* (*Neophlebotomus*) *gemmea* as vector of any *Leishmania* species from any other geographical areas. In fact, *Sergentomyia* species were rarely considered as vector of medically important *Leishmania* since they prefer animal blood. Only a few studies could detect *Leishmania* DNA in *Sergentomyia*. A study in India identified *L. donovani* DNA in *S. babu*[20], while Parvizi & Amirkhani (2008) identified *L. major* DNA in *S. sintoni* in Iran [21]. Recently *L. major* DNA was also detected in *S.* (*Spelaeomyia*) *darlingi* collected from zoonotic cutaneous leishmaniasis affected areas in Mali [22]. Moreover, *L. major* was also isolated from *S. garnhami* and successfully cultured in NNN medium [23]. Since *Sergentomyia* sandflies are zoophilic, they might have a role in the transmission of zoonotic leishmaniasis. Leishmaniasis caused by *L. siamensis* might be also zoonotic since phylogenetic analysis showed that *L. siamensis* was closely related to *L. enrietti*, a *Leishmania* species infecting Brazilian guinea pigs [6]. In addition, *L. siamensis* DNA was identified in animals such as rats and dogs (personal communication).

The distribution of sandfly species in the present study was similar to our previous report showing that *S.* (*Neophlebotomus*) *gemmea* was predominant in southern Thailand, the affected area of leishmaniasis caused by *L. siamensis*[14]. Unfortunately, we could not detect *L. siamensis* by PCR in any samples of the previous survey [14]. Of the 26 species of sandflies described in different regions of Thailand [11], the distribution of species in the South was different from other parts of Thailand [11,14]. All captured *S.* (*Neophlebotomus*) *gemmea* were found outdoors where the cow corral was the most common habitat. Habitat preference could be related to their blood meal feeding. In this study, we identified *S.* (*Neophlebotomus*) *gemmea* based on morphological characteristics [15,16]. The 18s rRNA of *L. siamensis*-positive *S.* (*Neophlebotomus*) *gemmea* was amplified and sequenced. The nucleotide sequence was then submitted to GenBank. Unfortunately no other 18s rRNA nucleotide sequence of *S.* (*Neophlebotomus*) *gemmea* is available to be compared in GenBank. Since the chromatogram of nucleotide sequences showed a completed consensus, we postulated that each pool contained only one species.

## Conclusion

Our study showed that *S.* (*Neophlebotomus*) *gemmea* might be a vector of *L. siamensis*, the causative agent of leishmaniasis in Thailand*.* Further studies with larger samples are urgently needed to determine the potential sandfly vectors, their infection rate and behaviors such as host-preference behavior, blood meal feeding and breeding habitat in all affected areas of leishmaniasis. In addition, the natural reservoir host should be identified. The awareness of the disease by public health personnel and community health workers as well as having an effective vector control program are important keys to prevent the disease from spreading.

## Competing interests

The authors declare that they have no competing interests.

## Authors’ contribution

KK, PT, MM and SL designed the study protocol. UN, SK and PJ collected and determined species of sandflies. SS, AH and CC performed PCR tests. KK, SS, MM and SL drafted and revised the manuscript. All authors have given approval to the final version of the manuscript.

## Pre-publication history

The pre-publication history for this paper can be accessed here:

http://www.biomedcentral.com/1471-2334/13/333/prepub
